# Circulating Inflammatory and Mitochondrial Biomarkers Associated with Cachexia in Advanced Non-Small Cell Lung Cancer

**DOI:** 10.3390/cancers18040655

**Published:** 2026-02-17

**Authors:** Kamya Sankar, Elham Kazemian, Nicole Lorona, Carlos D. Cruz-Hernández, Alex K. Bryant, Mitra Mastali, Akil A. Merchant, Jennifer Van Eyk, Karen L. Reckamp, Puneeth Iyengar, Neil A. Bhowmick, Jane C. Figueiredo

**Affiliations:** 1 Department of Medicine, Samuel Oschin Comprehensive Cancer Center, Cedars-Sinai Medical Center, Los Angeles, CA 90048, USA; 2 Department of Radiation Oncology, Veterans Affairs Ann Arbor Healthcare System, Ann Arbor, MI 48105, USA; 3 Department of Cardiology, Smidt Heart Institute, Cedars-Sinai Medical Center, Los Angeles, CA 90048, USA; 4 Advanced Clinical Biosystems Research Institute, Department of Biomedical Sciences, Cedars-Sinai Medical Center, Los Angeles, CA 90048, USA; 5 Department of Radiation Oncology, Memorial Sloan-Kettering Cancer Center, New York, NY 10065, USA

**Keywords:** cachexia, non-small cell lung cancer, mitochondrial DNA, biomarkers

## Abstract

**Background**: Cancer-associated cachexia is a multifactorial metabolic syndrome characterized by progressive skeletal muscle and/or adipose tissue loss and affects approximately 40% of patients with non-small cell lung cancer (NSCLC). However, reliable circulating biomarkers for early detection and risk stratification remain undefined. Based on prior observations linking elevated circulating mitochondrial DNA (mtDNA) to cachexia, we hypothesized that mtDNA and inflammatory protein levels in plasma could predict cachexia onset and trajectories. **Methods**: We evaluated 27 patients with stage IV NSCLC enrolled in the SeroNet-CORALE cohort with plasma samples collected between 2020 and 2023. Forty biomarkers were quantified at two timepoints (T1 and T2) using a multiplexed MesoScale Discovery platform. Associations between log2-transformed biomarker levels and cachexia status were assessed using Firth’s penalized logistic regression. **Results**: Among 27 patients (65% female; mean age 65 ± 10 years; 89% adenocarcinoma histology), cachectic patients exhibited lower body mass index at both time points (T1: 21.0 ± 2.0 vs. 27.0 ± 7.0; T2: 21.8 ± 4.9 vs. 25.2 ± 4.9). At T1, cachexia was strongly associated with elevated GDF15 (OR 4.29; 95% CI 1.04–29.74; *p* = 0.044) and IL-15 (OR 43.83; 95% CI 2.39–>999; *p* = 0.007), whereas IL-4 had a protective association (OR 0.09; 95% CI 0.00–0.66; *p* = 0.013). At T2, cachexia was associated with higher mtDNA levels (OR 2.13; 95% CI 1.07–7.69; *p* = 0.022) and lower levels of IL-15, IL12/IL23p40, and MDC. **Conclusions**: Distinct inflammatory and mitochondrial biomarkers tracked cachexia evolution in advanced NSCLC, with early GDF-15/IL-15 elevations and later increases in circulating mtDNA. Larger longitudinal studies are warranted to validate these findings and define their clinical relevance.

## Simple Summary

Cancer-associated cachexia is a common and serious condition in patients with advanced lung cancer, leading to unintentional weight loss, muscle wasting, and poor quality of life. Currently, there are no reliable blood-based biomarkers to identify patients who are at risk of developing cachexia. In this study, we analyzed blood samples from patients with stage IV non-small cell lung cancer to determine whether inflammatory proteins and mitochondrial DNA levels in the blood were associated cachexia development and progression. We found that cachectic patients exhibit distinct changes in the blood biomarkers at different stages of the disease. Early cachexia is associated with higher levels of inflammatory proteins GDF-15 and IL-15, while cachexia later in the disease course was associated with increased circulating mitochondrial DNA. Larger studies are needed to confirm these results and assess their clinical utility.

## 1. Introduction

Lung cancer remains the leading cause of cancer-related death worldwide despite substantial advances in therapies [1]. Cancer-associated cachexia is a major, yet frequently underrecognized, contributor to morbidity and mortality in this population. Cachexia is a complex metabolic syndrome characterized by profound alterations in body composition with progressive loss of skeletal muscle with or without adipose tissue depletion that cannot be fully reversed by conventional nutritional interventions [2,3,4,5]. These changes drive progressive functional decline, decreased treatment tolerance, diminished quality of life, and increased mortality. Cachexia affects 40–50% of patients with lung cancer [6,7] and contributes directly to mortality in up to 20% of cases [8,9].

Cachexia has been consistently associated with poor response to chemotherapy, earlier cessation of treatment, and markedly shortened survival [10]. By the time patients present with overt emaciation, they often exhibit the refractory stage of cachexia, during which therapeutic benefit is minimal and expected survival is less than three months [11]. Although cachexia is a well-established predictor of poor prognosis, it is frequently diagnosed only after patients have already progressed to advanced, irreversible stages. Validated circulating biomarkers to identify patients at high risk or to detect early, potentially reversible phases of the syndrome are currently lacking. Earlier detection of cachexia may allow for timely intervention, inform treatment strategies, and potentially improve survival outcomes.

Mitochondrial dysfunction has been broadly implicated in contributing to skeletal muscle wasting in cancer-associated cachexia. Early and progressive mitochondrial dysfunction in skeletal muscle is associated with reduced mitochondrial biogenesis and ATP production, leading to energy deficits and muscle wasting [12,13]. These mitochondrial changes are not limited to skeletal muscle but also affect adipose tissue and liver, disrupting whole-body energy homeostasis and amplifying the cachectic phenotype [14]. Mitochondrial DNA (mtDNA), which plays a key role in regulating mitochondrial function, becomes compromised early in cachexia. Circulating mtDNA may therefore reflect early mitochondrial damage and serve as a minimally invasive biomarker of emerging mitochondrial dysfunction. In parallel, multiple inflammatory pathways—most notably IL-6, CRP, and the cachexia-inducing cytokine GDF-15 [15]—have been implicated in the pathophysiology of cancer-associated cachexia, supporting the value of evaluating a broad panel of systemic inflammatory and metabolic markers alongside mitochondrial DNA.

In this prospective cohort study, we investigated the relationship between plasma biomarkers, including circulating mtDNA along with a broad panel of cytokines, chemokines, metabolic hormones, and angiogenic factors, for their risk of cachexia in patients with advanced non-small cell lung cancer. We aim to identify candidate plasma biomarkers that enable earlier detection, improve risk stratification, and inform future therapeutic interventions.

## 2. Materials and Methods

### 2.1. Patient Cohort

**Figure 1:**
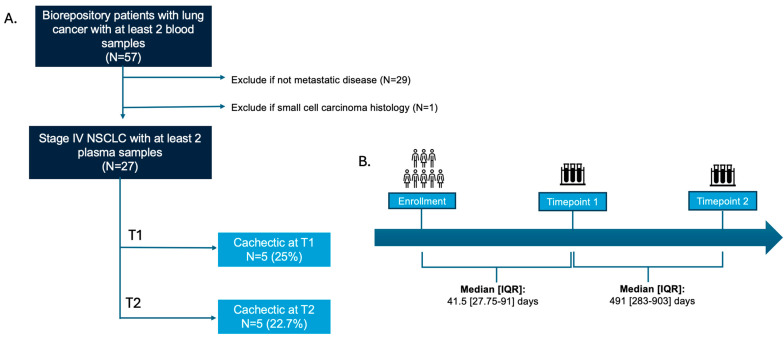
(**A**) Study Cohort and Sample Selection Flow Diagram. Flow diagram depicting cohort selection from participants enrolled in the U.S. National Cancer Institute (NCI)-funded cohort study (U54CA260591). The final cohort included 27 patients with stage IV non-small cell lung cancer who had at least 2 plasma samples available. Cachexia prevalence was 25% at T1 and 22.7% at T2. (**B**) Study Timeline and Blood Collection Schema. Schematic representation of study timepoints and the interval between blood collections. Blood was obtained at enrollment, T1 and T2. The median time from enrollment to T1 was 41.5 days (Interquartile range (IQR): 27.75–91), and the median interval between T1 and T2 was 491 days (IQR: 283–903).

We included individuals with a diagnosis of stage IV non-small cell lung cancer (ICD-10 code category: C34) who were participating in a U.S. National Cancer Institute (NCI)-funded cohort study (U54CA260591). This is a prospective longitudinal cohort study enrolling patients aged 18 or older with histologically confirmed malignancies who are under active anticancer treatment. Patients were recruited from 3 November 2020 to 31 March 2023. In this study, eligibility criteria included adults (18 years) with histologically confirmed stage IV non-small cell lung cancer receiving active anti-cancer therapy and with at least two plasma samples available for biomarker profiling (Figure 1A). The sample size was determined by the availability of participants in the SeroNet-CORALE cohort with stage IV NSCLC and at least two plasma samples during the study period. No formal exclusion criteria were prespecified; however, all patients were clinically reviewed at enrollment and were confirmed to have no concurrent second primary malignancy or severe hepatic or renal dysfunction. Regulatory approval was obtained from the Cedars-Sinai Institutional Review Board and the USAMRDC Office of Human Research Oversight. All participants provided written informed consent.

### 2.2. Data Collection

Electronic medical records (EMRs) were queried using the Cedars-Sinai Enterprise Information System (EIS) to extract detailed demographic, clinical, treatment, laboratory, and pathology data for all enrolled participants. Data capture was designed to include nearly all clinically available information relevant to cancer diagnosis, treatment, and outcomes.

Demographic data included date of birth, age (at diagnosis and at each encounter), sex assigned at birth, race, ethnicity (Hispanic vs. non-Hispanic), address, city, state, ZIP code, country, and primary phone number. Vital status (alive/deceased) and most recent contact date were recorded, along with identifiers such as social security number and primary care provider to enable accurate linkage across data systems.

Clinical data were derived from patient encounters and included diagnosis name and date, corresponding ICD-9 and ICD-10 codes, first and most recent record of diagnosis, and encounter-level information such as encounter type (inpatient, outpatient, emergency department), admission and discharge dates, discharge disposition, and attending provider. Tumor-specific variables included cancer site, histologic classification, grade, and stage (clinical and pathologic). ECOG performance status, immunization history, and weight and vital sign trajectories were captured from longitudinal flowsheet data.

Procedural and treatment data incorporated both inpatient and ambulatory procedures, surgical reports, and billing data (OpTime, CPT, and ICD-9 procedure codes). Detailed anti-cancer treatment regimens were extracted, including medication name, class, dosage, route, start and stop dates, infusion rates (for inpatient therapies), and treatment intent. Pharmacy data captured all systemic therapies (chemotherapy, immunotherapy, hormonal therapy), as well as supportive medications, with order identifiers, quantities, and refills.

Laboratory and biomarker data were drawn from institutional lab systems. They included complete blood count (CBC), metabolic panels, and other relevant assays, with timestamps for specimen collection, processing, and result reporting. Each result included the specimen source and type, result value, and units. Tumor molecular and pathology data were obtained from institutional reports, capturing tumor grade, margin status, recurrence (if applicable), and molecular profiling results from next-generation sequencing (NGS). Biomarker data included programmed death ligand-1 (PD-L1) tumor proportion score, mismatch repair (MMR) or microsatellite instability (MSI) status, and other clinically relevant mutations (e.g., *EGFR*, *KRAS*, *ALK*, *MET*). Additional data domains included social and family history (e.g., smoking and alcohol use, substance use, sexual activity, family history of cancer or other diseases), allergies (agent, reaction, severity, and date noted), and imaging data (order identifiers, procedure codes, impression/narrative text). Pathology and biospecimen tracking were integrated from the institutional biobank, including accession numbers, specimen source and type, collection and processing timestamps, and associated result comments. All data were cross validated for accuracy and completeness. Automated EIS extraction was supplemented by targeted manual chart review by trained study staff and medical physicians to adjudicate missing, ambiguous, or conflicting information.

### 2.3. Defining Cachexia

Cachexia was defined according to the international consensus criteria (i.e., (i) weight loss of 5% or more within the past 6 months, or (ii) weight loss of 2% or more when body weight index (BMI) was less than 20 kg/m^2^ within the past 6 months. Cachexia status was assessed at diagnosis (T0) and at two additional time points (Timepoint 1 (T1) and Timepoint 2 (T2)) during the disease course, corresponding to the timing of available plasma samples (Figure 1B). The timing of T1 and T2 was determined by biospecimen availability rather than prespecified intervals, consistent with the pragmatic design of the cohort; cachexia status was therefore assessed contemporaneously at each time point to account for variability in disease progression and treatment course. When objective pre-diagnosis weight data were unavailable, clinical progress notes were independently reviewed by a board-certified medical oncologist to determine whether clinical documentation supported the presence of cachexia at diagnosis (e.g., charted weight loss, functional decline, reduced oral intake, or evidence of muscle wasting).

### 2.4. Biomarker Analysis

Venous blood samples were collected and processed in the Institutional BioBank and Research Pathology Core Laboratory following standardized protocols. Plasma (from ACD and EDTA tubes), buffy coat, and peripheral blood mononuclear cells were isolated by centrifugation and Ficoll density separation, aliquoted into cryovials and cryopreserved. All specimens were barcoded, de-identified and stored under controlled conditions at −80 °C in accordance with institutional biosafety procedures. Biomarkers were assessed at T1 and T2. In cases where more than two blood samples were available, T2 was designated as the sample collected at the greatest interval from T1 (Figure 1B).

The following markers were evaluated using Meso Scale Discovery (MSD): Angiogenic markers (V-PLEX)—βFGF, PIGF, sFlt-1, Tie-2, VEGF, VEGF-C, VEGF-D; Cytokine panel—IL12/IL23p40, IL-15, IL-16, IL-17, IL-1α, IL-5, IL-7, TNF-β, VEGF; Inflammatory panel—IFN-γ, IL-10, IL-13, IL-1β, IL-2, IL-4, IL-6, IL-8, TNF-α; Markers of cachexia—GDF-15, mitochondrial DNA; Chemokines—Eotaxin, IP-10, MCP-1, MCP-4, MDC, MIP-1α, MIP-1β, TARC; Metabolic markers—insulin, leptin and Vascular injury markers—CRP, SAA, sICAM-1, sVCAM-1. The calibrators were prepared according to the manufacturer protocol. Samples were diluted with the recommended diluent and dilution factor for each panel. Plate was washed with 150 µL of wash buffer (1× PBS/0.05%Tween20) three times and tap dry. For each panel the volume recommended for samples and calibrators were pipetted in each well. The plate was sealed and placed on a shaker for 2 h at room temperature. Plates were washed and detection mix prepared according to the manufacturer protocol was pipetted in all well. After the 2 h incubation, the plate was washed three times with wash buffer and tap dry. To read the plate on MSD instrument, the MESO QuickPlex SQ 120, 150 µL of 2× Read Buffer (supplied in the kit), was pipetted in each well. The calculations to establish calibration curves and determine concentration and data analysis were carried out using MSD Workbench 4.0 software. The software fits the standard curves using a 4-parameter logistic fit with 1/y2 weighting.

Plasma was also analyzed to measure mitochondrial DNA. The QIAamp minielute ccfDNA kit (QIAgen, Hilden, Germany, cat. no. 55204) was used to process 500 µL of human plasma for ccfDNA purification and mtDNA quantification. Then, plasma was mixed with 30 µL magnetic bead-suspension, 55 µL proteinase K and 150 µL bead binding buffer. The following steps were performed according to the manufacturer instructions, after bead elution, the protocol was completed using a QIAcube connect device (QIAgen, cat. no. 9002864). For mtDNA quantification, a standard curve (X = log2(x)) was prepared measuring COX2, a human mitochondrial gene, by real-time PCR with mtDNA isolated from PC3 cells. The implemented formula to calculate mtDNA concentrations (ng/mL) contained in plasma was x = 2^((Ct−b)/m)^ × 1000.

### 2.5. Statistical Analysis

Patient demographic, clinical, and treatment variables were harmonized across timepoints (diagnosis, T1, T2). Continuous variables were summarized as mean ± standard deviation and categorical variables as counts and percentages. Prior to analysis, biomarker concentrations below the limit of detection were imputed as LLOD/$\sqrt{2}$, and all clinical variables were verified for completeness via manual chart review; no biomarker data points were missing from the analyzed samples. Characteristics were reported overall and stratified by cachexia status at T1 and T2, with group comparisons using chi-square or Fisher’s exact tests for categorical variables and *t*-tests for continuous variables. Cachexia prevalence at diagnosis, T1, and T2 was summarized as counts and proportions and visualized with annotated bar plots. Longitudinal weight trajectories were plotted for each participant, highlighting cachectic individuals, with summary statistics reported for each timepoint. Associations between circulating biomarkers and cachexia were assessed at each timepoint using Firth penalized logistic regression to account for small-sample bias and rare events. Baseline (T1) biomarkers were evaluated for their association with cachexia at 6 months and at T2. Biomarker levels were log2-transformed, and models were adjusted for age, sex, and race. Odds ratios (OR) with 95% confidence intervals (CI) and *p*-values were reported, with extremely small (<0.001) or large (>999) ORs noted. Given the exploratory nature of this biomarker discovery study and the limited sample size, we report unadjusted *p*-values to avoid excessive type II errors that could obscure potentially important biological signals. Candidate biomarkers were then pre-selected based on significant associations (*p* < 0.05) with cachexia status using Firth logistic regression were compared between cachectic and non-cachectic participants using violin and boxplots, with differences assessed by Wilcoxon rank-sum tests and FDR-adjusted for multiple comparisons. We evaluated associations between high plasma mtDNA levels (defined as ≥median in cachectic participants) and elevated inflammatory biomarkers (defined as ≥overall cohort median) using Fisher’s exact tests. Nine biomarkers representing diverse inflammatory pathways were analyzed: CRP (acute phase), IL-15, IL-6 (pro-inflammatory), IL-5, IL-4 (anti-inflammatory/Th2), IL-12/IL-23p40 (Th1/Th17), MDC (chemokine), VEGF-C (angiogenesis), and sVCAM-1 (adhesion). All analyses were performed in R version 4.3.2 (31 October 2023 ucrt).

## 3. Results

### 3.1. Patient and Clinical Characteristics

A total of 27 patients with stage IV NSCLC were included (mean age 65 ± 10 years; 65% female). The cohort was predominantly composed of patients with adenocarcinoma histology (89%), with the remaining patients classified as squamous cell carcinoma (4%), not otherwise specified (4%), or other carcinoma (4%). Most patients had adenocarcinoma histology (89%), were White (59%), and were former or never smokers (52% and 41%, respectively) (Table 1). Baseline characteristics did not differ significantly between cachectic and non-cachectic patients at either time point. At the first time point (T1), 5 patients (19%) met criteria for cachexia. Cachectic patients had a lower BMI compared with non-cachectic patients (21.0 ± 2.0 vs. 27.0 ± 7.0), though this difference did not reach statistical significance (*p* = 0.13). Baseline demographics and clinical characteristics did not differ significantly between cachectic and non-cachectic patients at either time point, including age, sex, race/ethnicity, histology, PD-L1 expression, ECOG performance status, and treatment exposures. Although all cachectic patients at T1 harbored *EGFR*-mutant tumors (5/5), this did not match statistical significance given the small sample size (*p* = 0.10). 5 patients did not have molecular data available. ECOG performance status and treatment exposures (chemotherapy, targeted therapy, immunotherapy) were similar between cachectic and non-cachectic groups.

At the second time point (T2), 5 patients (19%) again met criteria for cachexia. 3 of these patients harbored *EGFR* mutations. BMI remained lower among cachectic patients (21.8 ± 4.9 vs. 25.2 ± 4.9; *p* = 0.20). Age, sex, race, smoking status, PD-L1 expression, histology, and oncogenic driver distributions did not differ significantly. ECOG scores trended toward worse performance status among cachectic patients, but this was not statistically significant (*p* = 0.20). Treatment patterns at T2—including chemotherapy (*p* = 0.20), targeted therapy (*p* = 0.57), and immunotherapy (*p* > 0.90)—were similar across groups. Overall, no demographic or clinical characteristics were significantly associated with cachexia at either time point.

### 3.2. Longitudinal Weight Trajectories

**Figure 2:**
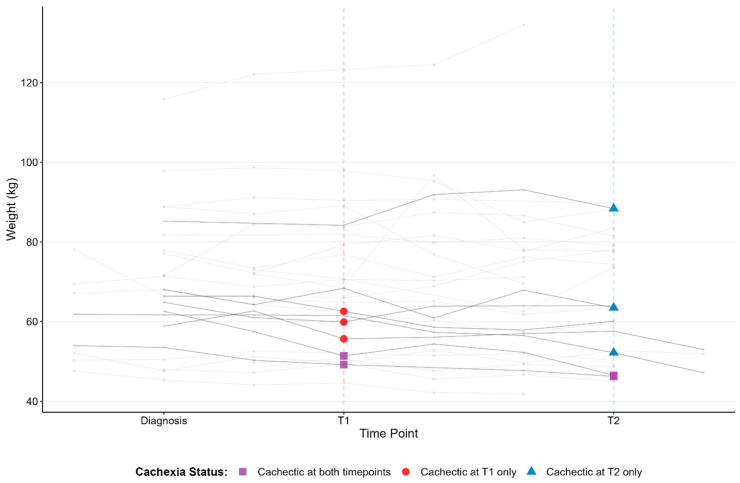
Longitudinal weight trajectories from diagnosis through T1 and T2 in patients with advanced NSCLC. Individual patient weight measurements are plotted from diagnosis to T1 and T2. Each line represents a single patient’s weight trajectory, with darker lines representing patients who developed cachexia at one or both collection timepoints. Vertical dashed lines indicate the timing of biospecimen collection at T1 (red) and T2 (blue). This figure illustrates the heterogeneity of weight change over time and highlights individuals who developed cachexia despite baseline weight and trajectory patterns. N = 27 patients; 5 patients were cachectic at T1, 5 patients were cachectic at T2, and 3 patients were cachectic at both time points.

To characterize the patterns of weight change over time, we examined individual patient weight trajectories from diagnosis through the two plasma collection timepoints (Figure 2). Across the cohort, weight patterns were heterogenous, with many patients experiencing modest fluctuations and a subset demonstrating more pronounced weight decline over time. Patients who met criteria for cachexia at T1 generally exhibited lower absolute body weight at that timepoint and showed evidence of progressive decline from diagnosis to T1. In contrast, individuals who developed cachexia only at T2 often demonstrated relative weight stability early in their disease course, followed by substantial weight loss between T1 and T2. Three patients who were cachectic at T1 improved and/or stabilized their weight subsequently.

Two patients met cachexia criteria at both timepoints and showed the most consistent downward trajectories, reflecting persistent weight loss across the study interval. Patients who remained non-cachectic showed a wide range of weight patterns, including both stability and mild decline, but did not exhibit the steep reductions observed in cachectic individuals.

### 3.3. Biomarkers of Interest

Firth penalized logistic regression was used to evaluate associations between circulating biomarkers and cachexia at each timepoint, adjusting for age, sex, and race. Several biomarkers demonstrated significant and time-dependent relationships with cachexia status (Table 2).

**Figure 3:**
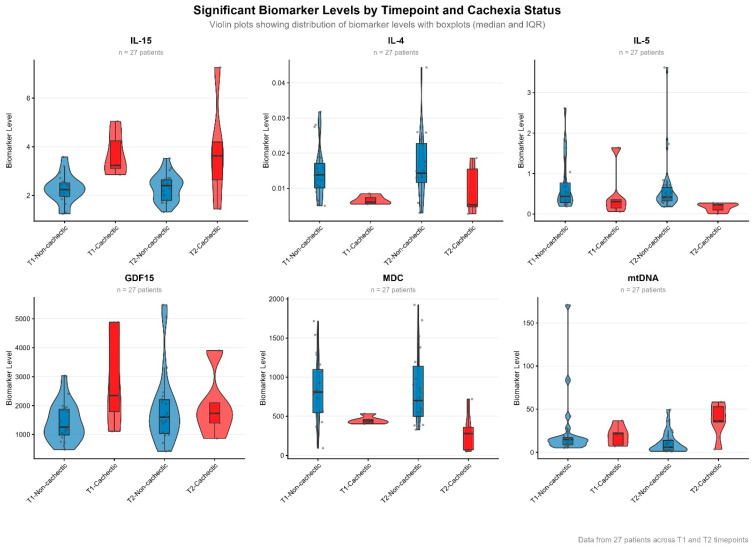
Differences in biomarker levels between groups were examined using box-and-violin plots, which display both the median and interquartile range (box), as well as the full distribution of values (violin).

At T1, three biomarkers showed strong associations with cachexia. GDF-15 significantly elevated in cachectic patients (OR 4.29, 95% CI: 1.04–29.74, *p* = 0.044). IL-15 demonstrated the strongest association at this early timepoint, with markedly higher levels in cachectic patients (OR 43.83, 95% CI: 2.39–>999, *p* = 0.007; Figure 3). Conversely, IL-4 exhibited a protective association, with lower levels linked to increased odds of cachexia (OR 0.09, 95% CI: 0.00–0.66, *p* = 0.013). In contrast, different biomarkers emerged at T2. Circulating mitochondrial DNA was significantly higher in patients with cachexia at the later timepoint (OR 2.13, 95% CI: 1.09–7.69, *p* = 0.022). By T2, IL-15 levels no longer showed a significant positive association with cachexia (OR 4.66, *p* = 0.04). IL-5 (OR 0.17, 95% CI: 0.00–0.083, *p* = 0.011), macrophage derived chemokine (MDC) (OR 0.26, 95% CI: 0.02–0.74, *p* = 0.006) and IL-12/IL-23p40 (OR 0.44, 95% CI: 0.15–0.84, *p* = 0.010) showed protective effects against cachexia, with IL-4 still suggesting a protective effect (OR 0.33, 95% CI: 0.07–1.06), *p* = 0.063; Figure 3).

### 3.4. Association of Biomarkers with Subsequent Development of Cachexia

To determine whether circulating biomarkers measured at T1 could predict future onset or progression of cachexia, we evaluated associations between T1 biomarker levels and (1) cachexia status at 6 months following T1 and (2) cachexia status at the subsequent biospecimen collection timepoint (T2). Firth logistic regression models were adjusted for age, sex, and race (Table 3). Higher MCP-1 (*p* = 0.070) and higher eotaxin (*p* = 0.072) measured at T1 trended towards association of cachexia at 6 months following T1. However, none surpassed the threshold of statistical significance for early biomarker changes at T1 to reliably predict short-term cachexia at a 6-month interval. In contrast, several biomarkers measured at T1 were significantly associated with cachexia status at T2. Two immunomodulatory chemokines were significantly associated with lower odds of developing cachexia at T2. Higher T1 levels of MDC were associated with a significantly reduced risk of cachexia at T2 (OR 0.19, 95% CI: 0.02–0.77, *p* = 0.016). Elevated T1 levels of TARC were similarly protective against cachexia at T2 (OR 0.28, 95% CI: 0.04–0.96, *p* = 0.041). Additionally, sVCAM-1 showed a trend towards reduced cachexia risk (*p* = 0.083). Notably, no biomarkers were significantly associated with increased risk of developing cachexia at T2.

### 3.5. Change in Biomarker Levels Between T1 and T2 and Association with Cachexia at T2

To determine whether dynamic changes in circulating biomarkers could more accurately reflect evolving cachexia, we evaluated the change in biomarker levels from T1 to T2 and cachexia status at 2 using Firth logistic regression, adjusted for age, sex, and race (Table 4).

Four biomarkers demonstrated statistically significant associations with cachexia at T2. An increase in IL-15 (OR 6.36, 95% CI: 1.09–101.60, *p* = 0.0387) and circulating mtDNA (OR 2.18, 95% CI: 1.15–9.12, *p* = 0.00977) between T1 and T2 were independently associated with higher odds of cachexia at T2. In contrast, an increase in IL-4 (OR 0.23, 95% CI: 0.03–0.08, *p* = 0.0173) and IL-12/IL-23p40 (OR 0.32, 95% CI: 0.06–0.82, *p* = 0.0117) between T1 and T2 were independently associated with reduced cachexia risk at T2. Several biomarkers demonstrated near-significant trends including IL-5 (OR 0.57, 95% CI: 0.22–1.02, *p* = 0.0597) and MDC (OR 0.49, 0.08–1.26, *p* = 0.144) but did not reach statistical significance.

## 4. Discussion

In this prospective cohort study of patients with advanced NSCLC, we found that elevated circulating mtDNA was associated with both the presence of cachexia at a given time point and the subsequent development of cachexia. Importantly, changes in mtDNA levels between serial blood collections were also associated with cachexia progression, highlighting the potential utility of mtDNA as a candidate biomarker of both early detection and monitoring of this debilitating syndrome in patients with cancer. Multiple studies have previously demonstrated that mitochondrial dysfunction is an early and central event in cancer cachexia [12,13,14,15]. Brown et al. (2017) demonstrated that mitochondrial degeneration precedes muscle atrophy in tumor-bearing mice, suggesting a causative role rather than a consequential event [16]. Our findings build upon prior work implicating mitochondrial dysfunction in cancer-associated cachexia, suggesting that circulating mtDNA may reflect early and ongoing mitochondrial injury contributing to skeletal muscle wasting and systemic energy imbalance [15,17,18].

Consistent with prior studies [4], we observed associations between cachexia and well-established inflammatory mediators, including IL-15 and GDF-15 at the early time point T1. Although IL-15 has anabolic properties and correlates with less weight loss [19], it may also promote tumor growth and metastasis [20]. GDF-15, a known driver of cancer cachexia, induces anorexia and weight loss through central nervous system pathways, with higher levels correlating to severity [21,22]. Our data also suggested a nuanced interplay between pro- and anti-inflammatory pathways over the disease course. While pro-inflammatory cytokines showed strong associations with cachexia early in the course, several immunomodulatory cytokines, including IL-4, IL-5, MDC, and TARC, were associated with lower odds of cachexia, both cross-sectionally and in longitudinal analyses. While these findings suggest a dynamic balance between inflammatory and immunoregulatory pathways during cachexia progression, the present study was not designed to establish causality or define underlying immune mechanisms. Th2 cell mediated signaling has been implicated in exerting protective effects against muscle wasting and systemic inflammation [23,24]. Future mechanistic and longitudinal studies integrating immune phenotyping, muscle mass assessments, and mitochondrial injury markers will be necessary to clarify these mechanisms.

Our study has several clinical implications. First, circulating mtDNA may serve as a minimally invasive biomarker for risk stratification, enabling early identification of patients likely to develop cachexia. Second, the protective association of certain immunomodulatory cytokines raises the possibility of targeting these pathways therapeutically to modulate the inflammatory milieu and slow muscle wasting. Finally, integrating mitochondrial and cytokine biomarkers may provide a more comprehensive approach to monitoring disease progression and evaluating emerging therapeutic interventions to reduce and reverse cachexia. Interestingly, the consistent link between rising circulating mtDNA and later cachexia development extended beyond traditional inflammatory markers, even in patients not initially cachectic. This raises the possibility that mitochondrial dysfunction could reflect ongoing or nascent tissue wasting and may function alongside, rather than just downstream of, inflammation. Mechanistically, mitochondrial dysfunction impairs ATP production, alters reactive oxygen species balance, and promotes proteolysis in skeletal muscle [25,26], which may amplify tissue wasting independent of or alongside systemic inflammatory signals. Our findings suggest that early elevations of circulating mtDNA may identify patients at risk of progressive cachexia before overt clinical manifestations, offering a window for timely therapeutic intervention.

Several limitations should be acknowledged. First, the timing of plasma collection at T1 and T2 were determined by specimen availability within the SeroNet-CORALE protocol rather than prespecified milestones. The interval between study enrollment, T1, and T2 varied across participants (Figure 1B). To address this variability in disease course and cohort entry, cachexia status was defined contemporaneously at each sampling timepoint, enabling timepoint-specific characterization of biomarker associations despite non-uniform longitudinal intervals. Second, the sample size is modest and within a single institution, and validation in larger, independent, prospective cohorts is needed. Third, although the observed associations link circulating mtDNA and select cytokines with cachexia development and progression, the observational nature of this study precludes definitive causal inference. Lastly, as an exploratory study with analysis of multiple biomarkers, our findings are subject to potential false discovery. We have reported unadjusted *p*-values to maximize sensitivity for detecting candidate signals in this small cohort, recognizing that validation in larger studies is essential to confirm these associations. Future studies with a larger sample size incorporating longitudinally prespecified sampling, functional assessments of mitochondrial activity and muscle composition, and biomarker-guided interventional trials will be essential to establish mechanistic relevance and inform therapeutic potential.

In summary, our findings support a dual-pathway model of cancer-associated cachexia in advanced NSCLC, in which early inflammatory cytokines contribute to cachexia onset, while mitochondrial dysfunction, reflected by circulating mtDNA, may be a driver of progressive muscle wasting. Longitudinal biomarker profiling, including mtDNA and select immunomodulatory cytokines, may facilitate earlier detection, risk stratification, and ultimately offer personalized therapeutic interventions to mitigate the profound morbidity and mortality associated with cachexia in lung cancer. These findings require larger prospective studies to define their translational and clinical relevance.

## Figures and Tables

**Table 1:** Patient characteristics at diagnosis and stratified by cachexia status at collection time points T1 and T2.

	Overall	By Cachexia at T1			By Cachexia at T2		
Characteristic	N = 27 ^1^	Cachectic N = 5 ^1^	Not Cachectic N = 20 ^1^	*p*-Value ^2^	Cachectic N = 5 ^1^	Not Cachectic N = 22 ^1^	*p*-Value ^3^
Age (yrs)	65 ± 10	71 ± 10	68 ± 10	>0.9	71 ± 7	70 ± 11	0.9
Sex				0.6			0.6
Female	17 (65%)	4 (80%)	12 (63%)		4 (80%)	13 (62%)	
Male	9 (35%)	1 (20%)	7 (37%)		1 (20%)	8 (38%)	
Race/Ethnicity				0.6			0.5
Hispanic	2 (7.4%)	1 (20%)	1 (5.0%)		1 (20%)	1 (4.5%)	
White	16 (59%)	4 (80%)	11 (55%)		4 (80%)	12 (55%)	
Black	1 (3.7%)	0 (0%)	1 (5.0%)		0 (0%)	1 (4.5%)	
Asian	3 (11%)	0 (0%)	3 (15%)		0 (0%)	3 (14%)	
Other/Unknown	5 (19%)	0 (0%)	4 (20%)		0 (0%)	5 (23%)	
Smoking Status				0.8			0.8
Current Smoker	2 (7.4%)	0 (0%)	2 (10%)		0 (0%)	2 (9.1%)	
Former Smoker	14 (52%)	2 (40%)	11 (55%)		2 (40%)	12 (55%)	
Never Smoker	11 (41%)	3 (60%)	7 (35%)		3 (60%)	8 (36%)	
BMI	25.4 ± 6.3	21 ± 2	27 ± 7	0.13	21.8 ± 4.9	25.2 ± 4.9	0.2
Histology				>0.9			>0.9
Adenocarcinoma	24 (89%)	5 (100%)	17 (85%)		5 (100%)	19 (86%)	
Non-Small Cell Carcinoma, NOS	1 (3.7%)	0 (0%)	1 (5.0%)		0 (0%)	1 (4.5%)	
Squamous Cell Carcinoma	1 (3.7%)	0 (0%)	1 (5.0%)		0 (0%)	1 (4.5%)	
Other Carcinoma	1 (3.7%)	0 (0%)	1 (5.0%)		0 (0%)	1 (4.5%)	
PD-L1 Category				>0.9			>0.9
<1	6 (50%)	1 (100%)	4 (40%)		2 (67%)	4 (44%)	
1–50	4 (33%)	0 (0%)	4 (40%)		1 (33%)	3 (33%)	
50–100	2 (17%)	0 (0%)	2 (20%)		0 (0%)	2 (22%)	
Oncogene Driver				0.10			0.7
*ALK*	3 (11%)	0 (0%)	2 (10%)		0 (0%)	3 (14%)	
*EGFR*	10 (37%)	5 (100%)	4 (20%)		3 (60%)	7 (32%)	
*KRAS*	4 (15%)	0 (0%)	4 (20%)		1 (20%)	3 (14%)	
*MET*	2 (7.4%)	0 (0%)	2 (10%)		0 (0%)	2 (9.1%)	
No Driver	3 (11%)	0 (0%)	3 (15%)		1 (20%)	2 (9.1%)	
Not Tested	5 (19%)	0 (0%)	5 (25%)		0 (0%)	5 (23%)	
ECOG				0.6			0.2
0	11 (52%)	1 (20%)	9 (47%)		0 (0%)	2 (20%)	
1	8 (38%)	3 (60%)	7 (37%)		3 (60%)	8 (80%)	
2	1 (4.8%)	1 (20%)	3 (16%)		1 (20%)	0 (0%)	
3	1 (4.8%)				1 (20%)	0 (0%)	
Any Treatment	14 (52%)	5 (100%)	15 (75%)	0.5	5 (100%)	15 (68%)	0.3
Chemotherapy	9 (33%)	4 (80%)	13 (65%)	>0.9	4 (80%)	9 (41%)	0.2
Targeted Therapy	4 (15%)	2 (40%)	3 (15%)	0.3	1 (20%)	8 (36%)	0.6
Immunotherapy	8 (30%)	2 (40%)	12 (60%)	0.6	2 (40%)	8 (36%)	>0.9

^1^ Mean ± SD; n (%); ^2^ Wilcoxon rank sum exact test; Fisher’s exact test; Wilcoxon rank sum test; ^3^ Wilcoxon rank sum exact test; Fisher’s exact test; Overall characteristics measured at diagnosis. T1: First collection time point; T2: Second collection time point. All variables are timepoint-specific (age, BMI, ECOG, and treatments measured at respective timepoints). Treatment categories are not mutually exclusive (patients may receive multiple treatment types). Abbreviations: BMI = Body Mass Index; PD-L1 = Programmed Death-Ligand 1; ECOG = Eastern Cooperative Oncology Group Performance Status.

**Table 2:** Association between inflammatory biomarkers and cachexia at the first and second timepoints using logistic regression models.

	Timepoint 1		Timepoint 2	
Biomarker	Model *	*p*-Value	Model *	*p*-Value
CRP	0.64 (0.24–1.27)	0.207	0.56 (0.22–1.03)	0.065
Eotaxin	3.92 (0.97–26.23)	0.056	1.44 (0.45–5.51)	0.541
Eotaxin-3	0.89 (0.23–3.08)	0.840	1.11 (0.29–4.33)	0.873
GDF15	4.29 (1.04–29.74)	**0.044**	1.02 (0.32–3.21)	0.976
IFN-γ	1.50 (0.74–3.41)	0.259	0.86 (0.36–1.80)	0.693
IL-10	1.99 (0.93–5.01)	0.075	1.05 (0.42–2.55)	0.908
IL-13	0.03 (<0.001–1.13)	0.059		
IL-15	43.83 (2.39–>999)	**0.007**	4.66 (0.79–75.86)	0.094
IL-16	0.46 (0.13–1.30)	0.141	0.71 (0.24–2.06)	0.518
IL-17	1.02 (0.46–2.18)	0.964	1.01 (0.35–2.09)	0.979
IL-1α			1.00 (0.04–13.08)	0.998
IL-1β	0.75 (0.43–1.23)	0.250	0.94 (0.41–1.99)	0.859
IL-2	1.81 (0.59–8.96)	0.315	0.62 (0.16–2.15)	0.440
IL-4	0.09 (0.00–0.66)	**0.013**	0.33 (0.07–1.06)	0.063
IL-5	0.64 (0.22–1.48)	0.303	0.17 (0.00–0.83)	**0.011**
IL-6	1.00 (0.37–1.57)	0.986	1.06 (0.51–1.61)	0.804
IL-7	1.30 (0.48–3.70)	0.598	2.55 (0.86–13.56)	0.095
IL-8	0.87 (0.37–1.84)	0.715	0.56 (0.17–1.44)	0.236
IP-10	1.99 (0.80–8.84)	0.144	0.39 (0.04–1.85)	0.248
IL12/IL23p40	0.98 (0.30–3.12)	0.967	0.44 (0.15–0.84)	**0.010**
Insulin	1.43 (0.74–3.22)	0.289	1.26 (0.64–2.58)	0.501
Leptin	0.95 (0.60–1.44)	0.822	1.11 (0.74–1.68)	0.584
MCP-1	1.63 (0.36–10.17)	0.523	0.49 (0.06–3.25)	0.453
MCP-4	0.36 (0.06–1.37)	0.139	0.37 (0.06–1.45)	0.160
MDC	0.46 (0.10–1.46)	0.194	0.26 (0.02–0.74)	**0.006**
MIP-1α	2.04 (0.63–8.04)	0.228	0.47 (0.05–2.04)	0.465
MIP-1β	5.47 (0.91–85.54)	0.064	1.60 (0.30–10.23)	0.579
PIGF	1.49 (0.14–14.36)	0.718	0.80 (0.11–2.15)	0.677
SAA	1.05 (0.51–2.05)	0.895	1.04 (0.69–1.50)	0.822
TARC	0.82 (0.29–2.03)	0.673	0.29 (0.04–1.02)	0.054
TNF-α	2.56 (0.71–13.60)	0.147	0.18 (0.01–1.41)	0.111
TNF-β	0.26 (<0.001–3.01)	0.303	1.97 (0.64–9.78)	0.251
Tie-2	6.48 (0.49–716.97)	0.177	1.76 (0.16–23.34)	0.640
VEGF-C	0.51 (0.05–2.10)	0.370	0.13 (0.00–1.03)	0.054
VEGF-D	1.69 (0.23–22.76)	0.621	0.69 (0.08–5.19)	0.717
VEGF.x	1.34 (0.26–9.95)	0.730	0.41 (0.07–1.71)	0.226
VEGF.y	0.50 (0.15–1.15)	0.110	0.74 (0.15–1.60)	0.480
bFGF	0.88 (0.50–1.37)	0.576	2.16 (0.85–8.03)	0.112
mtDNA	0.93 (0.35–2.22)	0.865	2.13 (1.09–7.69)	**0.022**
sFlt-1	1.74 (0.79–7.85)	0.174	1.42 (0.81–2.78)	0.223
sICAM-1	12.57 (0.82–658.11)	0.070	0.62 (0.05–4.66)	0.647
sVCAM-1	4.28 (0.42–76.64)	0.222	0.08 (<0.001–1.61)	0.107

* Model was adjusted for sex, age at diagnosis, and race. Biomarker levels were log2-transformed prior to analysis. Firth logistic regression was applied to reduce small-sample bias. Odds ratios (OR) and 95% confidence intervals (CI) are reported, with extreme values shown as ‘<0.001’ or ‘>999’. Abbreviations: IFN-γ, interferon gamma; IL, interleukin; TNF, tumor necrosis factor; CRP, C-reactive protein; ICAM, intercellular adhesion molecule 1; SAA, serum amyloid A; VCAM, vascular cell adhesion molecule. Bolded *p*-value indicates statistical significance.

**Table 3:** Associations between biomarkers at Time point 1 and cachexia at 6 months after Time point 1 and cachexia at Time point 2.

	6 Months After Timepoint 1		Timepoint 2	
Biomarker	Model *	*p*-Value	Model *	*p*-Value
CRP	0.46 (0.11–1.04)	**0.065**	0.65 (0.24–1.30)	0.233
Eotaxin	3.03 (0.92–18.30)	0.072	1.89 (0.47–8.51)	0.361
Eotaxin-3	1.33 (0.48–3.84)	0.57	1.00 (0.26–3.58)	0.996
GDF15	0.34 (0.06–1.23)	0.103	0.91 (0.21–3.49)	0.89
IFN-γ	1.49 (0.86–3.10)	0.159	1.20 (0.59–2.39)	0.592
IL-10	1.37 (0.70–3.50)	0.364	0.99 (0.31–2.13)	0.979
IL-13	NA (NA–NA)	NA	NA (NA–NA)	NA
IL-15	0.84 (0.13–4.97)	0.842	0.56 (0.06–4.46)	0.579
IL-16	1.02 (0.38–2.85)	0.972	0.68 (0.26–1.75)	0.403
IL-17	1.17 (0.62–2.25)	0.622	0.89 (0.37–1.90)	0.767
IL-1α	NA (NA–NA)	NA	NA (NA–NA)	NA
IL-1β	1.11 (0.68–2.02)	0.67	1.17 (0.70–2.23)	0.559
IL-2	0.81 (0.31–2.08)	0.653	0.97 (0.32–3.22)	0.963
IL-4	0.91 (0.25–3.05)	0.879	2.04 (0.51–11.44)	0.316
IL-5	1.72 (0.83–4.16)	0.147	0.69 (0.23–1.64)	0.397
IL-6	0.91 (0.53–1.35)	0.647	1.20 (0.70–1.96)	0.429
IL-7	0.71 (0.22–1.83)	0.494	1.33 (0.50–3.77)	0.559
IL-8	1.56 (0.79–3.55)	0.202	1.07 (0.48–2.30)	0.855
IP-10	1.33 (0.72–2.84)	0.359	0.95 (0.29–2.24)	0.902
IL12/IL23p40	0.64 (0.18–1.86)	0.421	0.53 (0.13–1.70)	0.293
Insulin	0.99 (0.50–1.96)	0.981	1.28 (0.66–2.76)	0.46
Leptin	0.82 (0.52–1.19)	0.308	1.04 (0.69–1.57)	0.828
MCP-1	3.72 (0.91–42.70)	0.0702	2.54 (0.50–24.97)	0.28
MCP-4	2.45 (0.79–11.38)	0.125	0.45 (0.08–1.67)	0.236
MDC	0.51 (0.15–1.44)	0.206	0.19 (0.02–0.77)	**0.0157**
MIP-1α	0.69 (0.21–1.76)	0.44	0.27 (0.02–1.40)	0.136
MIP-1β	0.93 (0.25–3.26)	0.902	0.79 (0.11–4.21)	0.796
PIGF	0.26 (0.02–1.85)	0.183	0.51 (0.03–4.98)	0.585
SAA	0.71 (0.31–1.37)	0.32	1.02 (0.52–1.93)	0.952
TARC	0.88 (0.35–2.07)	0.764	0.28 (0.04–0.96)	**0.041**
TNF-α	1.32 (0.49–4.66)	0.58	0.85 (0.03–3.25)	0.849
TNF-β	0.00 (<0.001–1.03)	**0.0523**	1.89 (0.09–491.88)	0.667
Tie-2	0.38 (0.03–3.22)	0.372	0.85 (0.09–8.87)	0.881
VEGF-C	1.05 (0.27–3.80)	0.94	0.47 (0.06–1.85)	0.305
VEGF-D	0.99 (0.17–7.03)	0.989	0.22 (0.02–1.57)	0.131
VEGF.x	0.61 (0.13–2.52)	0.491	0.67 (0.11–3.66)	0.639
VEGF.y	1.05 (0.47–2.39)	0.895	0.92 (0.41–2.00)	0.831
bFGF	0.89 (0.53–1.34)	0.577	1.05 (0.66–1.64)	0.838
mtDNA	0.62 (0.22–1.35)	0.24	0.55 (0.14–1.33)	0.202
sFlt-1	0.40 (0.06–1.37)	0.208	1.10 (0.42–2.47)	0.818
sICAM-1	0.42 (0.03–4.33)	0.467	0.51 (0.03–5.87)	0.592
sVCAM-1	0.29 (0.02–2.22)	0.238	0.09 (0.00–1.33)	0.0834

* Model was adjusted for sex, age at diagnosis, and race. Biomarker levels were log2-transformed prior to analysis. Firth logistic regression was applied to reduce small-sample bias. Odds ratios (OR) and 95% confidence intervals (CI) are reported, with extreme values shown as ‘<0.001’ or ‘>999’. Abbreviations: IFN-γ, interferon gamma; IL, interleukin; TNF, tumor necrosis factor; CRP, C-reactive protein; ICAM, intercellular adhesion molecule 1; SAA, serum amyloid A; VCAM, vascular cell adhesion molecule; NA, not available. Bolded *p*-value indicates statistical significance.

**Table 4:** Associations between changes in inflammatory biomarkers and cachexia outcome at second measurement using Firth’s penalized logistic regression.

Biomarker	Model *	*p*-Value
CRP	0.67 (0.27–1.26)	0.245
Eotaxin	0.92 (0.30–3.01)	0.888
Eotaxin-3	1.01 (0.40–3.24)	0.991
GDF15	1.12 (0.33–3.85)	0.843
IFN-γ	0.80 (0.30–1.46)	0.506
IL-10	1.12 (0.43–4.16)	0.842
IL-15	6.36 (1.09–101.60)	0.0387
IL-16	1.10 (0.43–2.91)	0.83
IL-17	1.06 (0.51–2.33)	0.871
IL-1β	0.79 (0.40–1.31)	0.385
IL-2	0.41 (0.04–2.30)	0.323
IL-4	0.23 (0.03–0.80)	0.0173
IL-5	0.57 (0.22–1.02)	0.0597
IL-6	0.47 (0.07–1.48)	0.229
IL-7	1.36 (0.62–4.56)	0.472
IL-8	0.71 (0.31–1.40)	0.324
IP-10	0.79 (0.33–1.97)	0.556
Il12/Il23p40	0.32 (0.06–0.82)	**0.0117**
Insulin	0.96 (0.51–1.84)	0.906
Leptin	1.24 (0.60–3.10)	0.574
MCP-1	0.40 (0.08–1.44)	0.163
MCP-4	0.87 (0.27–2.95)	0.816
MDC	0.49 (0.08–1.26)	0.144
MIP-1α	1.33 (0.37–5.69)	0.677
MIP-1β	2.10 (0.39–22.72)	0.467
PIGF	0.87 (0.16–2.78)	0.819
SAA	1.05 (0.61–1.76)	0.831
TARC	1.08 (0.44–3.10)	0.866
TNF-α	0.41 (0.05–2.42)	0.314
TNF-β	4.67 (0.70–>999)	0.127
Tie-2	6.82 (0.13–531.03)	0.334
VEGF-C	0.51 (0.06–2.04)	0.351
VEGF-D	2.78 (0.40–30.02)	0.312
VEGF.x	0.73 (0.21–2.10)	0.568
VEGF.y	0.88 (0.43–1.58)	0.668
bFGF	1.18 (0.72–2.43)	0.528
mtDNA	2.18 (1.15–9.12)	**0.00977**
sFlt-1	1.57 (0.81–3.60)	0.186
sICAM-1	0.86 (0.08–12.08)	0.901
sVCAM-1	1.23 (0.12–31.58)	0.87

* Model was adjusted for sex, age at diagnosis, and race. Biomarker levels were log2-transformed prior to analysis. Firth logistic regression was applied to reduce small-sample bias. Odds ratios (OR) and 95% confidence intervals (CI) are reported, with extreme values shown as ‘<0.001’ or ‘>999’. Abbreviations: IFN-γ, interferon gamma; IL, interleukin; TNF, tumor necrosis factor; CRP, C-reactive protein; ICAM, intercellular adhesion molecule 1; SAA, serum amyloid A; VCAM, vascular cell adhesion molecule. Bolded *p*-value indicates statistical significance.

## Data Availability

The data that support the findings of this study are available from the corresponding author upon reasonable request.
